# Feasibility study of the Cambridge knee injury tool (CamKIT): 18-month analysis of acute knee injuries at an urgent treatment centre^☆^

**DOI:** 10.1016/j.jcot.2025.103203

**Published:** 2025-09-09

**Authors:** Thomas Molloy, Benjamin Gompels, Simone Castagno, Andrew McCaskie, Stephen McDonnell

**Affiliations:** a Division of Trauma and Orthopaedic Surgery, University of Cambridge, Addenbrooke's Hospital, Cambridge, CB2 0QQ, UK; b Faculty of Medicine, University of Queensland, Mayne Medical School, 20 Weightman St, Herston, QLD, 4006, Australia

**Keywords:** Knee, Injury, Risk, Prediction, Clinical

## Abstract

**Background:**

Soft tissue knee injuries are associated with many short- and long-term consequences. Currently, obstacles in the diagnostic pathway lead to inefficiencies in the management process, resulting in suboptimal patient outcomes. Early stratification of knee injury severity is essential for guiding timely investigations and management decisions. This study aimed to prospectively evaluate how the Cambridge Knee Injury Tool (CamKIT) performs in stratifying knee injuries at initial presentation.

**Methods:**

This prospective observational study involved eighty-five participants presenting with acute knee injuries in a Major Regional Urgent Treatment Centre (UTC) over 18 months. Patients recorded information regarding patient factors, external factors, injury mechanisms and signs and symptoms at index presentation. CamKIT scores were then calculated. Patients then continued the management stream determined by the treating healthcare team. Management decisions were not influenced by the results of the CamKIT score. Patient outcomes were analysed by extracting time-to-event outcomes, injury classification, and management information. Statistical analysis involved sensitivity and specificity calculations, descriptive statistics, and Mann-Whitney Test calculations between injured and non-injured cohorts, and surgical and conservative cohorts.

**Results:**

The tool achieved a sensitivity of 100 % and specificity of 31.7 %, with a negative predictive value (NPV) of 100 % and a positive predictive value (PPV) of 34.9 %. CamKIT scores were higher in patients with injuries (median 8; IQR: 7–9) than in non-injured patients (median 6; IQR: 4–7) (p < 0.0001). Similarly, patients who proceeded to surgical intervention (n = 9) had higher scores (median 8; IQR: 7.5–10) compared to those managed conservatively (median 6; IQR: 4.25–7) (p = 0.0001).

**Conclusion:**

The CamKIT continues to show promise in guiding early triage and management of acute knee injuries. Future research involves a multicenter study as well as the integration of machine learning to develop a more robust prediction tool and create a risk-stratified management protocol.

## Graphical abstract

**Figure:**
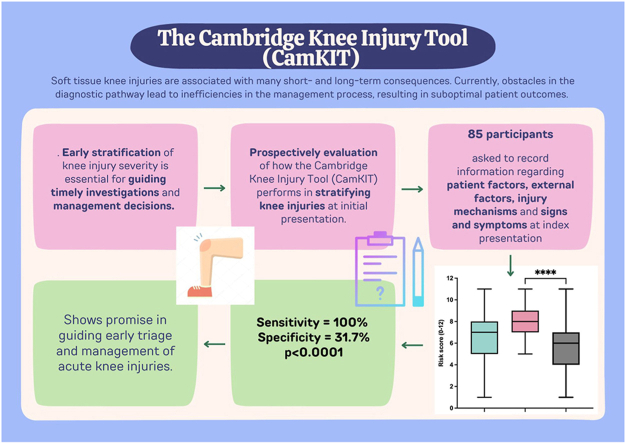

## 1 Introduction

Knee injuries are among the most common musculoskeletal complaints encountered in emergency departments, primary care, and sports medicine clinics.^1^, ^2^, ^3^ They represent a significant proportion of acute musculoskeletal presentations, ranging from benign soft tissue sprains to clinically significant ligamentous, meniscal, or osteochondral damage, which may require early specialist input, advanced imaging, or surgical intervention.^4^^,^^5^

Timely and accurate identification of severe knee pathology at the initial presentation remains a significant clinical challenge.^6^^,^^7^ Current diagnostic pathways rely on non-specific clinical signs, variable practitioner experience, and limited access to gold standard diagnostic imaging.^7^^,^^8^ These barriers contribute to inefficient care, prolonged physical and mental patient symptoms, and potential long-term sequelae such as joint instability, chronic pain, and early onset post-traumatic osteoarthritis.^9^, ^10^, ^11^

Existing clinical decision tools, such as the Ottawa Knee Rules and Pittsburgh Knee Rules, primarily aim to identify fractures and guide radiographic use.^12^ However, few validated instruments exist to risk-stratify soft tissue injuries and guide the urgency of further assessment or intervention. Currently, other tools screen solely for ACL injury, which limits their clinical utility in primary care settings.^5^^,^^13^^,^^14^ As a result, clinicians often depend on subjective judgement, which may lead to inconsistent outcomes and resource utilisation.^15^

To address this, the Cambridge Knee Injury Tool (CamKIT) was developed as a novel, non-invasive risk stratification instrument designed to evaluate the severity of soft tissue knee injuries for triage by combining patient-reported factors and clinical findings available at the initial point of contact (Table 1).^15^ It aims to offer structured guidance on the likelihood of significant pathology and support evidence-based decisions regarding imaging and referrals. This study aims to evaluate the performance of the CamKIT tool in stratifying knee injury severity at the time of initial presentation.

**Table 1:** The twelve variables and their scorings used for risk calculation in the model. Each variable carried a binary weighting of 0 or 1.

Variables	Answer (Score)
1. Patient medical history of an ipsilateral STKI	Yes (1)/No (0)
2. Type of sport or activity during injury.	High risk (1)/Low risk (0)
3. Mechanism	Non-contact (1)/Contact (0)
4. Swelling of the injured knee.	Yes (1)/No (0)
5. Reported rapid swelling of the knee.	Yes (1)/No (0)
6. Inability to weight bear.	Yes (1)/No (0)
7. Significant reduction in range of movement	Yes (1)/No (0)
8. Reported twisting or pivoting of the knee during injury.	Yes (1)/No (0)
9. Reported hyperextension of the knee during injury.	Yes (1)/No (0)
10. Reported feeling of any instability, “giving-way”, or “shifting” in the injured knee.	Yes (1)/No (0)
11. Reported sound or feeling of any “popping”, “cracking” or “tearing” in the knee during injury.	Yes (1)/No (0)
12. Reported sound or feeling of any “locking”, “catching”, or “clicking” in the injured knee.	Yes (1)/No (0)

## 2 Methods

This prospective observational study was conducted over 18 months from October 2023 to March 2025 in the Urgent Treatment Centre (UTC) and the Trauma and Orthopaedics Clinic at Addenbrooke's Hospital, Cambridge, United Kingdom. The study population comprises individuals aged 16 years and older who present with a primary complaint of an acute knee injury. Inclusion criteria for the study are: patients aged over 16 years, presenting with an acute knee injury sustained within the last 72 h, and attending the UTC. Patients were excluded if they were under 16 years of age, presented with a non-acute knee injury (i.e. more than 72 h since injury), or had confirmed bony injuries. Additional exclusion criteria include the presence of neurovascular compromise, suspected knee dislocations, clinical intoxication at the time of presentation, multiple injuries, or if the patient is considered vulnerable due to the traumatic nature of the injury, such as cases requiring safeguarding or psychosocial intervention. A total of 85 patients presenting with acute knee injuries at the time of their index presentation were recruited.

At the point of initial assessment, patients completed a digital questionnaire on REDCap, capturing patient-related, external, and symptom-based variables (Appendix A). A CamKIT score ranging from 0 to 12 (Table 1) was automatically calculated and recorded. Each variable carried a binary weight of 0 or 1 (Table 1). Clinical care proceeded according to standard pathways, and treating clinicians were blinded to the CamKIT results to avoid influencing management decisions. The outcome of “soft tissue knee injury” was defined as the MRI-confirmed diagnosis of a grade one or two ligamentous injury or any meniscal injury. The CamKIT questionnaire has been previously validated in a retrospective cohort analysis.^15^

Patient outcomes were analysed retrospectively using electronic medical records (EMRs). The extracted data included MRI-confirmed injury classification and key time-to-event outcomes: time to fracture clinic appointment, MRI scan, and post-MRI consultant review. Final management decisions (surgical vs conservative) were also recorded.

Descriptive statistics were used to summarise baseline characteristics, reporting medians and interquartile ranges (IQRs) for continuous variables. Fischer's exact test was used to determine statistical significance for sex between the injured and non-injured cohorts, while the Mann-Whitney test was used to determine statistical significance for age. Time-to-event analyses were performed for key outcome metrics. A comparative study of CamKIT scores across injury subgroups was executed using the Mann-Whitney test, with a statistical significance threshold of p < 0.05. Statistical analysis was performed using GraphPad Prism version 10.1.0 for MacOS, GraphPad Software, Boston, Massachusetts, USA, www.graphpad.com.

This study was approved by the Cambridge University Hospitals NHS Foundation Trust Research and Development Department and received ethical approval from the Health Research Authority (HRA) and the Northern Ireland Research Ethics Committee (REC). The study was conducted under IRAS ID: 327031 and REC Reference: 23/NI/0136. Informed consent was obtained from all participants for the questionnaire and data use before their inclusion in the study. Data was stored securely on REDCap Safe Haven.^16^^,^^17^

## 3 Results

### 3.1 Demographics

Among the 85 patients, 52 (61 %) were male. Within the injured group, 15 out of 18 patients (83 %) were male. In the non-injured group, 37 out of the 67 (55 %) were male. There was a statistically significant difference between sexes in the injured and non-injured cohorts (p = 0.0329). The overall median age for patients (n = 83) was 32 years (interquartile range (IQR): 25–45). Injured patients (n = 18) had a median age of 29.5 years (IQR: 19.75–32.25), whereas non-injured patients (n = 65) had a median age of 33 years (IQR: 25–47). There was no statistical significance in age between the injured and non-injured cohorts (p = 0.0542).

### 3.2 Injury overview

Eighty-five patients were recruited, and eighteen (21 %) had confirmed injuries. Of the eighteen patients with MRI-confirmed injuries, only four (22 %) had isolated injuries. The majority had multi-structure involvement. The most common injuries included anterior cruciate ligament (ACL) tears (n = 11), followed by medial meniscus (n = 9), lateral meniscus (n = 8), medial collateral ligament (MCL) (n = 8), lateral collateral ligament (LCL) (n = 6), and a single posterior cruciate ligament (PCL) injury.

### 3.3 Management outcomes

**Fig. 1:**
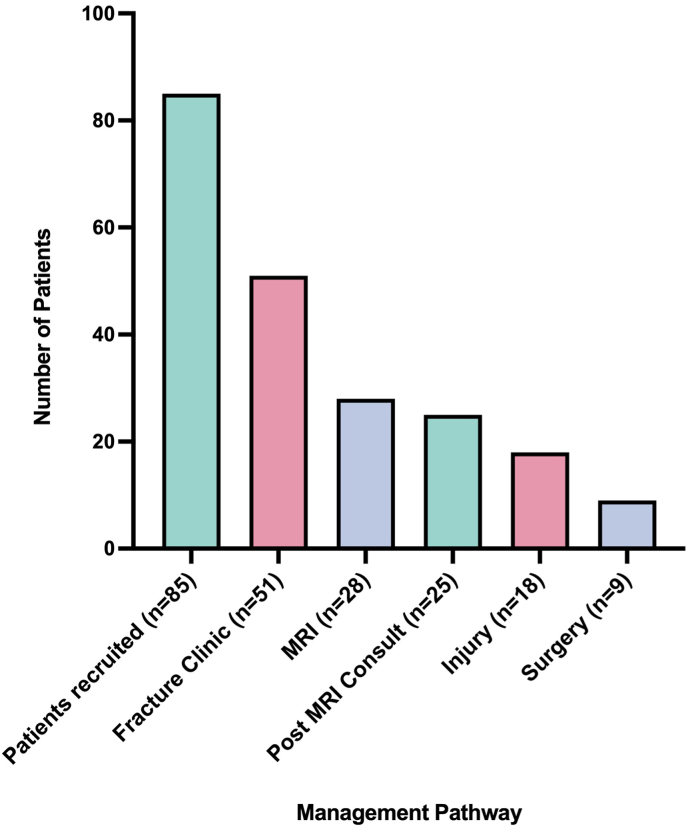
Proportion of injuries and surgeries among 85 patients.

Out of 85 recruited patients, 51 (60 %) attended a follow-up at the fracture clinic, and 28 (33 %) underwent MRI imaging. Among those who had an MRI, 25 (29 % of the total cohort) were reviewed in a post-MRI specialist consultation. Injuries confirmed by MRI were identified in 18 patients (21 %), and nine patients (11 %) ultimately required surgical intervention (Fig. 1).

### 3.4 Time-to-event analysis

**Fig. 2:**
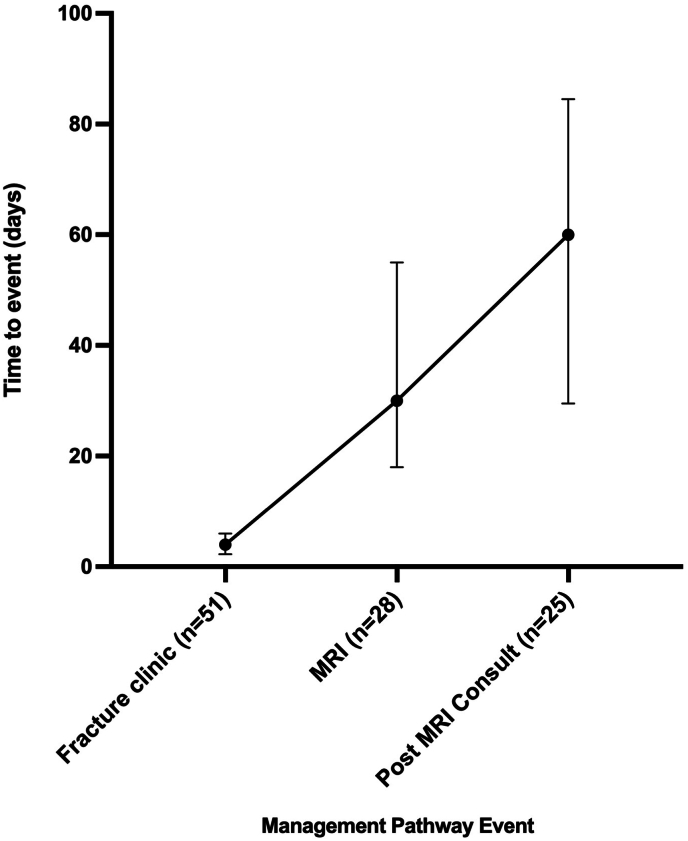
A bar chart showing the time taken to attend the fracture clinic, undergo magnetic resonance imaging (MRI), and attend specialist consultations (Median, IQR).

For the 52 patients in the fracture clinic cohort, the median time to appointment was 4 days (Interquartile range (IQR): 2.3–6 days). The median time for MRI scans (n = 28) was 30 days (IQR: 18–55). The post-MRI consultant review (n = 25) had a median wait time of 60 days (IQR: 30–85) (Fig. 2).

### 3.5 Diagnostic accuracy

The tool demonstrated a sensitivity of 100 % (95 % CI: 79.6–100) and a specificity of 31.7 % (95 % CI: 19.6–47) for detecting injury. The positive predictive value (PPV) was 34.8 % (95 % CI: 22.4–49.8), while the negative predictive value (NPV) was 100 % (95 % CI: 77.2–100) (Table 2).

**Table 2:** Contingency Table for Diagnostic Accuracy Calculations. Cut-off scores for risk categories are: Low 0–3, Medium 4–6, High 7–12).

Risk Category (Score)	Injury	No Injury
High (7–12)	15	28
Medium (4–6)	3	26
Low (0–3)	0	13

### 3.6 Injured vs non-injured

**Fig. 3:**
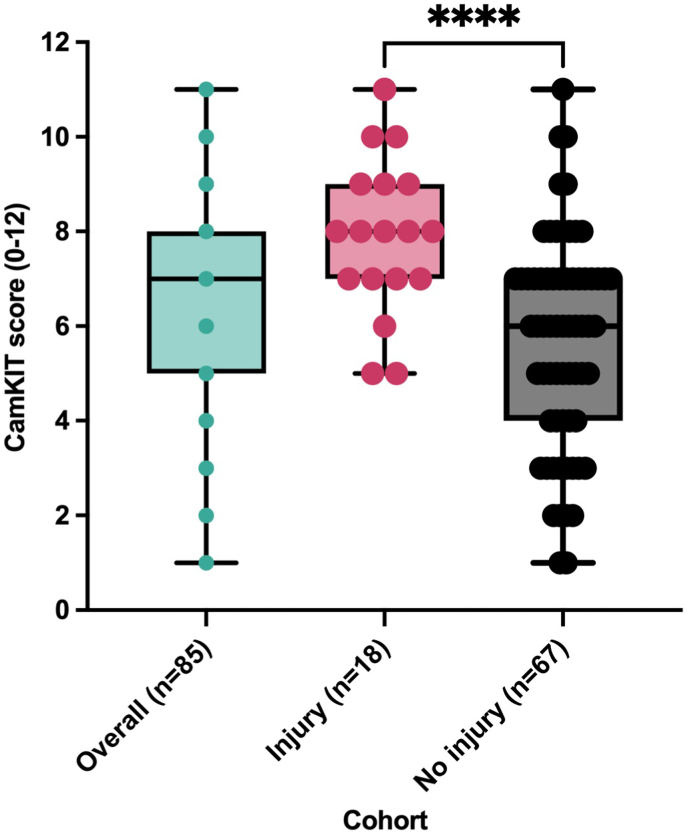
A box and whisker plot including all individual points indicating the comparative CamKIT Scores among injured and non-injured groups. Higher CamKIT scores correlated with MRI-confirmed injury.

Across all 85 patients, CamKIT scores ranged from 1 to 11. Patients with MRI-confirmed injuries (n = 18) had overall higher scores, with a median score of 8 (IQR: 7–9) compared to a median of 6 (IQR: 4–7) in the non-injury group (n = 67). The minimum score among injured patients was 5, while scores in the non-injury group ranged from 1 to 11. This is a statistically significant result of p < 0.0001 (Fig. 3).

### 3.7 Surgical vs conservative

**Fig. 4:**
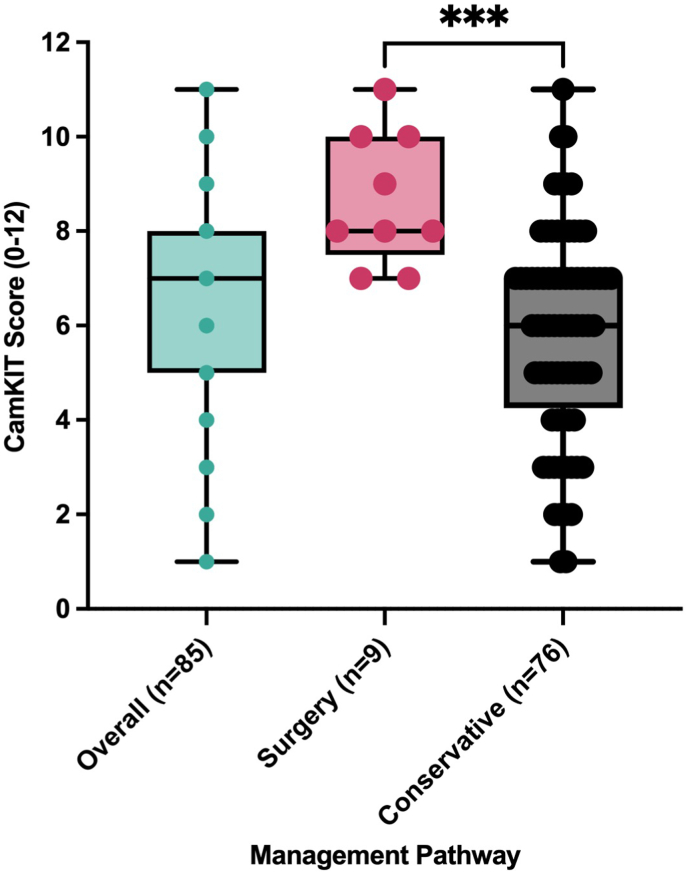
A box and whisker plot including all individual points indicating the CamKIT scores in surgical versus conservative patients. Higher CamKIT scores correlate with surgical management.

CamKIT scores were higher in patients who ultimately underwent surgical management (n = 9) than in those managed conservatively (n = 76). The surgical group had a median score of 8 (IQR: 7.5–10), while the conservative group had a median of 6 (IQR: 4.25–7). The minimum score in the surgical group was 7, indicating a higher baseline severity, while conservative cases ranged more broadly from 1 to 11. This is a statistically significant result of p = 0.0001 (Fig. 4).

## 4 Discussion

This study evaluated the CamKIT as a screening tool for patients with acute knee trauma. The findings support the tool's utility in stratifying injury severity at the time of initial presentation (Table 2). Patients with MRI-confirmed injuries had higher CamKIT scores than those without injuries (Fig. 3), and those who ultimately underwent surgery had the highest scores (Fig. 4). These results highlight CamKIT's capability to distinguish between injury severities and its potential role in guiding early management decisions.

The tool demonstrated a sensitivity of 100 % and a specificity of 31.7 % for detecting injury, with a negative predictive value of 100 % and a positive predictive value of 34.9 % (Table 2). These results support the high sensitivity observed in the previous development of the CamKIT and its role as a risk stratification tool. The lower specificity observed in this study may be attributed to increased recall bias, particularly when participants are presented with a comprehensive list of potential injury mechanisms and contextual factors. However, this is a recognised limitation in similar decision tools and does not undermine the clinical utility of maintaining high sensitivity. Notably, the diagnostic accuracy aligns with established tools such as the Ottawa Knee and Ankle Rules, meeting the critical threshold of 100 % sensitivity to ensure no injuries are missed. Clinically, this suggests that the tool could be effectively used as a triage threshold to discharge low-risk patients safely, reducing unnecessary fracture clinic attendances while maintaining patient safety. Despite its lower specificity, the identification of high-risk patients through the tool still provides an opportunity to expedite imaging and specialist referrals, ensuring that high-risk patients receive prompt and appropriate evaluation and management. Regarding issues of high false-positive rates, the Ottawa rules are widely implemented for referral guidance whilst demonstrating a similar specificity of 30–50 %, producing beneficial effects in significantly reducing unnecessary imaging.^12^^,^^18^

Strategies to improve specificity could include the adjustment of scoring thresholds into binary categories of high (7–12 CamKIT score) and low (0–6 CamKIT score). This would produce a sensitivity of 83 % and a specificity of 58 %. Whilst improving diagnostic specificity, this approach would hinder the tool's ability to confidently discharge patients in the low-risk category. This approach also limits the tool's utility to either discharging patients or referring them to an orthopaedic specialist, failing to account for patients in the medium risk category that may benefit from referral to physiotherapy services. Another strategy that has been utilised to improve specificity is through the integration of machine learning optimisation to utilise weighted scoring of variables. A robust machine learning analysis is a priority for future research.^19^

The further use of this tool in prehospital settings to rule out injuries has the potential to significantly reduce hospital presentations by providing clear guidance for managing low-risk injuries. The implementation of the tool in prehospital settings would require further robust validation of the tool and the implementation of an education program for pre-hospital healthcare providers and first responders about the tool's accurate application and its utility for management guidance. Its simplicity and ease of use mean that minimal training and education would be required for healthcare providers, trainers, and sports and exercise professionals, which could facilitate wider community and clinical uptake to improve early decision-making.

The tool also demonstrated relevance in clinical pathways: patients with higher scores were more likely to receive diagnostic imaging and surgical treatment, suggesting that CamKIT may assist clinicians in triaging cases and prioritising limited resources such as imaging slots or specialist reviews. The current median timeframes for fracture clinic (4 days), MRI (30 days), and post-MRI consultation (60 days) reflect typical delays in care, where a conclusive decision on management is not made until approximately 2 months after the time of injury (Fig. 2). These delays may be addressed through the use of an adjunct evidence-based management pathway that provides clinical guidance based on likely injury severity. In this area, existing soft tissue management pathways can be utilised to develop a simplified referral and management pathway for low, medium and high risk injury likelihood.^5^ Table 3 proposes a flowchart for how the CamKIT can streamline triage based on risk stratification. Furthermore, previous research has shown that expedited imaging with reserved time slots for high-risk musculoskeletal injury can reduce median time-to-MRI to 6.8 days.^20^ Consequently, this suggests that the expedited management of high-risk injuries may result in an improved time to clinical consultation following the MRI, potentially addressing current delays in care. Improving the time to management decision may subsequently improve not only the physical and psychological burdens of STKIs but also the incidence of post-traumatic osteoarthritis following the early initiation of either surgical or conservative management.^10^^,^^20^^,^^21^

**Table 3:** Proposal for clinical pathway integration to streamline management of acute soft tissue knee injuries.

Risk Level	Differential Diagnosis	Referral Pathway	Management Pathway
Low Risk (CamKIT score: 0–3)	Mild soft tissue injury/Bone contusion	- Consider physiotherapy referral - No referral to Virtual Fracture Clinic (VFC) or musculoskeletal specialist required - No urgent imaging required - If symptoms persist: Follow-up in primary care (GP, physiotherapy) - If symptoms worsen: Return to A&E	- Conservative treatment (RICE: Rest, Ice, Compression, Elevation) - Patient education on injury management - Gradual return to activity
Medium Risk (CamKIT score: 4–6)	Possible ligamentous or meniscal injury, possible cartilage involvement	- Physiotherapy referral and escalate if necessary - Consider further imaging (repeat X-ray, MRI if persistent instability or locking) - If symptoms worsen: Return to A&E	- Conservative treatment (RICE) - Patient education on injury management - Mobilise as tolerated until further assessment (no strenuous activity) - Early functional rehabilitation - Consider prehabilitation as potential surgical candidate
High Risk (CamKIT score: 7–12)	Likely ligament rupture, severe meniscal injury, multi-ligamentous injury, or associated fractures	- Referral to F2F musculoskeletal specialist or VFC (rural) - Physiotherapy referral - Likely need for MRI and/or repeat X-ray if fracture still suspected - Consider urgent in-person consultation if significant instability or locked knee	- Continue conservative treatment (RICE) - Mobilise as tolerated until further assessment (no strenuous activity) - Early functional rehabilitation - Commence prehabilitation as a potential surgical candidate

A strength of this study is the self-reported nature of the data, completed by patients at initial presentation. This supports CamKIT's usability in pre-hospital settings, including hyper-acute scenarios such as courtside or pitchside assessments, where rapid, structured injury evaluation can be challenging yet critical. The ability for players, coaches, or first responders to use a standardised tool immediately after injury could facilitate earlier identification of severe cases and prompt appropriate referrals or investigations. Implementing a patient-reported design subsequently reduces the subjectivity of the tool by limiting the requirement of clinician assessment, which reduces the variability of clinical proficiency and experience in assessing musculoskeletal injuries. Approaching the development of the tool with this design allows the tool to be validated with a standardised approach to STKIs that encompasses the most important risk factors, injury mechanisms and clinical findings.^7^^,^^15^

While the study was conducted at a single centre and included a modest sample size, its prospective design and real-world application in a specialist orthopaedic setting provide valuable insights. A critical step in the further development of this tool involves a multicenter analysis to improve the generalisability of this tool. The lack of long-term follow-up data limits conclusions about patient outcomes beyond initial diagnosis and management. The small surgical cohort is a critical limitation inherent to the single-centre design and 18-month follow-up period, limiting conclusions about CamKIT's predictive value, highlighting the need for larger multicenter validation. However, the low rate of surgical management among total presentations reflects the potential inefficiencies in referral pathways and the subsequent need for improved diagnostic accuracy.

To reduce the risk of recall bias, a uniformly structured questionnaire was provided to all participants (Appendix A), incorporating clear definitions of clinical features and terminology. The survey was designed in accordance with ethical standards, ensuring all content was presented using lay terms understandable to a non-clinical audience. Data was collected during the index visit to the Urgent Treatment Centre (UTC), ensuring a short recall period between symptom onset and reporting. The questionnaire also included temporal anchors and contextual prompts to guide accurate recollection. Importantly, the questionnaire was completed by patients prior to knowing the clinical outcome of their injury, ensuring blinding to diagnosis and management decisions at the time of reporting. While the questionnaire has been retrospectively validated, future work should include pilot testing and assessment of inter-rater reliability of the questionnaire.

This study may be subject to selection bias, as the cohort was drawn from patients presenting to an urgent treatment centre (UTC), potentially excluding individuals with more severe injuries or from elite sporting teams who were referred directly to specialist care. As a result, the findings may not fully represent the broader spectrum of acute knee injuries. This may lead to an underrepresentation of severe injuries, which may cause the CamKIT's performance to differ in tertiary or sports-specific settings. However, it is representative of the target cohort of patients presenting to the emergency department. Additionally, the study does not capture long-term outcomes, limiting the ability to assess the tool's predictive value over time or its impact on recovery, management decisions, or re-injury risk. This is a consideration to be included in the design of future long-term multi-centre research.

Additionally, if the tool were applied in broader clinical settings, such as, courtside, specialist sports medicine clinics, or tertiary trauma centres, its performance might differ due to variations in injury severity, complexity, and patient demographics not represented in the UTC cohort. This could lead to reduced accuracy or generalisability, particularly in populations with high-performance demands or atypical injury mechanisms.

Regarding the adjustment of potential confounders of sex and age, due to the relatively small sample size in this study, performing logistic regression or subgroup analyses would significantly reduce statistical power and was therefore not feasible. Nonetheless, these variables remain important considerations for future research, particularly within the context of a larger, multicentre study where multivariable analyses can be more robustly performed. Despite this limitation, the findings offer valuable insight into the risk factors and likelihood of injury, highlighting trends that may inform clinical decision-making.

## 5 Conclusion

The CamKIT is the first tool to demonstrate clinical utility in the early risk stratification of all STKIs by combining patient-reported and clinical factors, with the potential to provide pre-hospital guidance on injury management and streamline hospital imaging and surgical referrals. Further development of the CamKIT will involve machine learning analysis to refine its predictive accuracy and identify the most informative response patterns. A longitudinal multicentre validation is critical to assessing the tool's generalisability across different clinical settings within a larger cohort of patients. These steps will support the development of an acute knee injury assessment protocol incorporating the CamKIT, which can be deployed across emergency departments, sports facilities, and primary care or community settings to improve early diagnosis and management of knee injuries.

## Informed Consent

Informed Consent has been obtained from patient or guardian for the study's participation and publication.

## Author contribution

**T.M.**: Methodology, Investigation, Formal Analysis, Writing – Original Draft.

**B.G.**: Methodology, Investigation, Formal Analysis, Writing – Review & Editing.

**S.C.**: Methodology, Investigation, Formal Analysis, Writing – Review & Editing.

**A.M.**: Supervision.

**S.M.**: Conceptualisation, Supervision, Guarantor.

## Ethics approval

The study was conducted under IRAS ID: 327031 and NHS HRA REC Reference: 23/NI/0136.

## Patient or public involvement

Patients or the public were not involved in the design, or conduct, or reporting, or dissemination plans of our research.

## Data sharing

The data supporting this study's findings are available from the corresponding author, TM, upon reasonable request.

## Funding

Funding was provided by the Gwen Fish Foundation Orthopaedic Charitable Trust, United Kingdom.

## Declarations of competing interest

None.
